# New horizons in treatment of osteoporosis

**DOI:** 10.1186/s40199-017-0167-z

**Published:** 2017-02-07

**Authors:** Ozra Tabatabaei-Malazy, Pooneh Salari, Patricia Khashayar, Bagher Larijani

**Affiliations:** 1 Diabetes Research Center, Endocrinology and Metabolism Clinical Sciences Institute, Tehran University of Medical Sciences, Tehran, Iran; 2 Endocrinology and Metabolism Research Center, Endocrinology and Metabolism Clinical Sciences Institute, Tehran University of Medical Sciences, Fifth floor, Dr.Shariati Hospital, North Kargar Ave, Tehran, 14114 Iran; 3 Medical Ethics and History of Medicine Research Center, Tehran University of Medical Sciences, Tehran, Iran; 4 Osteoporosis Research Center, Endocrinology and Metabolism Clinical Sciences Institute, Tehran University of Medical Sciences, Tehran, Iran; 5 Center for Microsystems Technology, Imec and Ghent University, Gent-Zwijnaarde, Belgium

**Keywords:** Osteoporosis, Treatment, Fractures, Antiresorptive, Anabolic agents, Personalized medicine, Plants

## Abstract

**Background:**

Prevalence of osteoporosis is increasing both in developed and developing countries. Due to rapid growth in the burden and cost of osteoporosis, worldwide, it seems reasonable to focus on the reduction of fractures as the main goal of treatment. Although, efficient pharmacological agents are available for the treatment of osteoporosis, there still remains a need to more specific drugs with less adverse effects.

**Main body:**

This review article provides a brief update on the pathogenesis, presenting current pharmacological products approved by the US Food and Drug Administration (FDA) or Europe, and also newer therapeutic agents to treat osteoporosis according to the clinical trial data available at PubMed, UpToDate, International Osteoporosis Foundation (IOF), and clinical practice guidelines. As well, the effect of combination therapy and recommendations for future research will be further discussed.

**Short conclusion:**

The use of current antiresorptive and anabolic agents alone or in combinations for the treatment of osteoporosis entails several limitations. Mainly, their efficacy on non-vertebral fracture reduction is lower than that observed on vertebral fracture. In addition, they have potential adverse events on long time usage. Development of newer agents such as cathepsin k inhibitor and strontium ranelate not only have increased the available options for treating osteoporosis, but also have opened doors of opportunity to improvements in the effective treatment. However, the high cost of new agents have restricted their usage in selective patients who are at high risk of fracture or whom failed response to first line treatment options. Thus, personalized medicine should be considered for future evaluation of genetic risk score and also for environmental exposure assessment. In addition to permanent attention to early diagnosis of osteoporosis and understanding of the pathophysiology of osteoporosis for novel approach in drug discovery, there seems a need to more well-designed clinical trials with larger sample sizes and longer duration on current as well as on newer agents. Also, continuous research on plant-derived components as the source of discovering new agents, and conducting more clinical trials with combination of two or more synthetic drugs, plants, or drug-plant for the treatment of osteoporosis are recommended.

**Graphical Abstract:**

Summary of treatment modalities for osteoporosis.
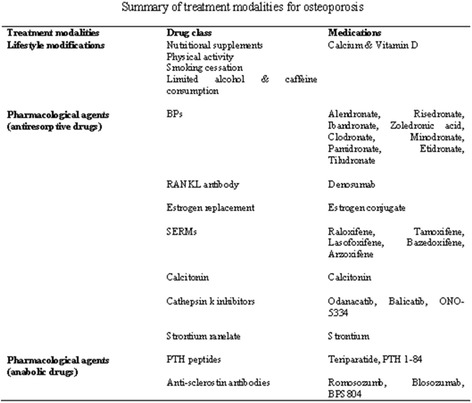

## Background

Osteoporosis is one of the common health problems with a progressive prevalence both in developed and developing countries [1–3]. The definition of Osteoporosis based on World Health Organization (WHO) criteria is reduction in bone mineral density (BMD) of 2.5 standard deviations or more below that of the mean peak BMD of young adults when measured by dual-energy x-ray absorptiometry (DEXA) [4]. This condition is influenced by different risk factors in terms of sex and age.

In accordance with the recent estimation of International Osteoporosis Foundation (IOF) there are 200 million osteoporotic women in the world with an accident of osteoporotic fracture every 3 seconds [1]. Both primary and secondary types of osteoporosis are associated with reduction in bone mass and microarchitecture that result in bone fragility with a significant increase in disability, morbidity, and fracture risk [5]. Thus, access to diagnosis and adequate therapy for osteoporosis is a main challenge worldwide.

Osteoporosis is more prevalent in women than men; 34% versus 17%, respectively. Usually, osteoporosis is prevalent in postmenopausal women and is presented as fractures in hip and spine due to accelerated bone turnover secondary to estrogen deficiency. However, vitamin D insufficiency and hyperparathyroidism remain as the main causes of osteoporosis in men and premenopausal women. By 2050, the worldwide incidence of hip fracture is expected to rise by 240% in women and 310% in men compared to 1990 involving approximately 1.66 million in 1990 to 6.26 million in 2050 [1]. Total cost for treating the prevalent osteoporotic-related fractures in the United States was estimated $19 billion in 2005 that would be tripled by the year 2040 [6, 7]. The annual hospitalization cost of osteoporotic fractures in US was shown to be equal or more than annual care costs associated with some chronic diseases such as myocardial infarction (MI), cerebrovascular accident, and breast cancer in 2011 [8].

A systematic review showed that the costs of osteoporosis treatment were not only greater than pre-fracture costs (as 1.6-6.2 times), but also were more than those spent for matched controls (2.2-3.5 times) [9].

Thus, due to the rapid growth in burden and cost of osteoporosis worldwide, it will be reasonable to focus on reduction of fractures as the main goal of treatment. WHO has developed an individual patient model known as FRAX® to calculate individual fracture risk identifying patients at higher risk of fracture based on clinical risk factors and femoral neck BMD [10]. In addition to this, it is important to have a more accurate and comprehensive look on the pharmacologic therapies of the osteoporosis.

This review article provides an update on the most current pharmacological products approved by Europe or the US Food and Drug Administration (FDA) to treat osteoporosis according to available data at PubMed, UpToDate, IOF, and clinical practice guidelines.

### Pathogenesis

Bone is composed of osteoblasts and osteocytes (bone-forming cells), osteoclasts (bone-reabsorbing cells) and osteoid (bone matrix). When the balance between bone resorption and deposition tips toward excessive resorption, bone loss occurs as a forerunner of osteoporosis [11].

Osteoporosis is a multifactorial disease which has a complex pathophysiology potentially caused by genetic, endocrine disorders, and nutritional factors. Some hormones have been shown to be effective in bone development among which parathyroid hormone (PTH), calcitonin, estrogen and vitamin D are considered the most important ones [12]. PTH not only can increase production of the activated form of vitamin D (1, 25-dihydroxyvitamin D or Calcidiol), but also can increase calcium absorption through kidneys, bone, and intestine. In addition, PTH can advance osteoclasts’ activity that result in further bone resorption. Calcitonin plays roles in protection of calcium by direct and indirect inhibition of the PTH effects that result in reduction of calcium resorption from kidneys, calcium uptake from intestine, and suppression of bone resorption. Calcitonin reversibly blocks osteoclasts’ function by binding to its receptor [12]. Estrogen reduces the rate of bone remodeling and increases osteoclast

apoptosis through two receptors including estrogen receptor α (ERα) and estrogen receptor β (ERβ). Estrogen deficiency has a critical role in the pathogenesis of osteoporosis due to its association with increased bone resorption, and impaired bone formation [13].

**Fig. 1:**
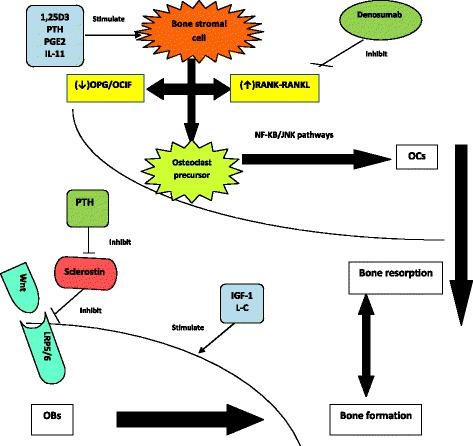
A brief schematic view of pathophysiology of osteoporosis and influence of some drugs on bone health. Legend: PTH: parathyroid hormone; PGE2: prostaglandin E2; IL-11: interleukin-11; OPG: osteoprotegerin; OCIF: osteoclastogenesis inhibitory factor; RANKL: receptor activator of nuclear factor κB ligand; NF-κB: nuclear factor κB; JNK: c-Jun N-terminal kinases; OCs: osteoclasts; Wnt: wingless and Int proteins; LRP5/6: LDL-receptor related protein 5/6; IGF-1: Insulin like growth factor-1; L-C: L-carnitine; OBs: osteoblasts

Bone micro-damages stimulate osteocytes to transmit resorption signals towards osteoclasts. In an immunological pathway, receptor activator of NF-κB (RANK) is expressed as pre-osteoclasts [14]. Function and differentiation of osteoclasts are regulated by RANK/RANK ligand (RANKL) interaction that can be blocked by osteoprotegerin (OPG) (Fig.1). PTH, PTH-related protein (PTHrP), cytokines, and prostaglandins can increase osteoclastogenesis by up-regulation of RANKL and down-regulation of OPG expression [12].

Bone remodeling requires the activities of both wingless-type and integrase 1 (Wnt) and bone morphogenetic protein (BMP) pathways to regulate osteoblast function (Fig.1). Critical role of Wnt signaling pathway, and absence of runt-related transcription factor 2 (Runx2) have been shown in regulating osteoblast differentiation and function. Wnt signaling is up-regulated by interaction of LDL receptor-related protein 5 (LRP5) and frizzled receptors (Frz), and is down-regulated by Dickkopf (DKK) as an inhibitor of LRP5 and secreted frizzled-related protein (SFRP) [13].

Sclerostin acts as an antagonist and blocks both BMP and Wnt signaling pathways, but canonical β-catenin performs as a synergism with BMP2 and Wnt. Thus, preventing mutations of gene encoding sclerostin (SOST) can increase bone formation and bone mass [8]. Some of other reasons associated with impaired bone formation are age-related reduction in the capacity of osteoblasts to replicate and differentiate, local and systemic growth factors deficiencies (IGF-1, TGF- β), prostaglandins (PGE2), leukotrienes (arachidonate 15-lipoxygenase that encoded by Alox 15), and leptin deficiency or resistance [13–15]. An inverse relationship between adipocytes and osteoblasts in the bone marrow via PPARγ has been shown as a master regulator of adipogenesis. Also, a close association not only between bone and insulin sensitivity, but also between osteoporosis and obesity or diabetes mellitus has been reported [16, 17].

The key role of oxidative stress, epigenetic, and gut microbiota in the pathogenesis of osteoporosis is established, too. Oxidative stress is an imbalance between the production of reactive oxygen species (ROS) and antioxidative defense. The effect of oxidative stress on bone is presented as reduction in bone mass, bone formation, osteoblast numbers and dysfunction of osteoblasts [16–19]. Epigenetic is referred to a stable change of normal gene expression without altering DNA sequence in response to various environmental stimuli, different pharmacological agents, and nutrients. All three types of epigenetic markers including DNA methylation, posttranslational histone modification, and miRNA are influenced by regulation of gene expression in bone cells [18, 20]. The effects of gut microbiota on bone cell have been mediated through immune systems. Gut microbiota can increase bone mass and density and can strengthen it by enhancing the synthesis and absorption of Ca, magnesium, vitamins K and B12. Moreover, the synthesis of serotonin and availability of tryptophan as a serotonin precursor, as well as suppression of osteoclastogenesis is improved [21].

### Management of osteoporosis

Pharmacological agents are used as the typical treatment of osteoporosis. However, lifestyle modifications through receiving adequate nutritional supplements (including calcium intake about 1200 mg daily and vitamin D intake in range of 1000-2000 IU daily with higher amount in some patients), weight bearing activity at least 30 minutes daily, avoiding or stopping smoking, avoiding heavy alcohol consumption to ≤2 servings daily, limiting caffeine intake, and practical and emotional support by health providers, family and friends have important roles in maintaining bone health [22–24]. In addition, surgical treatments such as vertebroplasty and kyphoplasty have been used for pain relief. It is notable that the main adverse effect of osteoporosis is the increasing risk of vertebral fracture. Due to limitations in published studies assessing the role of these procedures for osteoporosis, their benefits have still remained uncertain [22, 23]. Summary of recommended lifestyle modifications and pharmacological agents for osteoporosis are shown in Table 1.

**Table 1:** Summary of treatment modalities for osteoporosis

Treatment modalities	Drug class	Medications
Lifestyle modifications	Nutritional supplements Physical activity Smoking cessation Limited alcohol & caffeine consumption	Calcium & Vitamin D
Pharmacological agents (antiresorptive drugs)	BPs	Alendronate, Risedronate, Ibandronate, Zoledronic acid, Clodronate, Minodronate, Pamidronate, Etidronate, Tiludronate
	RANKL antibody	Denosumab
	Estrogen replacement	Estrogen conjugate
	SERMs	Raloxifene, Tamoxifene, Lasofoxifene, Bazedoxifene, Arzoxifene
	Calcitonin	Calcitonin
	Cathepsin k inhibitors	Odanacatib, Balicatib, ONO-5334
	Strontium ranelate	Strontium
Pharmacological agents (anabolic drugs)	PTH peptides Anti-sclerostin antibodies	Teriparatide, PTH 1-84 Romosozumb, Blosozumab, BPS804

Legend: *BPs* Bisphosphonates, *SERMs* selective estrogen receptor modulator

#### Calcium and vitamin D supplementation

In some countries calcitriol and alfa-calcidol have been used as synthetic analogues of vitamin D for the treatment of osteoporosis. A meta-analysis showed that vitamin D supplementation alone cannot reduce fracture risk. However, the results of another meta-analysis revealed a fracture risk reduction at vertebral and non-vertebral sites [25, 26]. However, the beneficial effect of calcitriol was reported for prevention of bone loss in osteoporosis after glucocorticoid therapy or after transplantation of solid organ or stem cell [27]. The main adverse effects of vitamin D derivatives are increasing the serum and urine level of calcium. The beneficial effects of adequate intake of calcium (Ca) and vitamin D on rate of bone loss and fracture risk has been shown in a study [28]. Some of meta-analysis studies reported 0.81-0.87 relative risk reduction for hip fracture (13-19% reduction) by combination of Ca with vitamin D [29, 30]. Generally, recommended daily intake of Ca and vitamin D in postmenopausal osteoporotic women is 1200 mg (total intake by diet and supplements) and 800 international units (IU), respectively. These amounts can change to 1000 mg (total intake by diet and supplements) and 600 IU, respectively, in premenopausal osteoporotic women and men [31].

#### Pharmacological agents for treatment of osteoporosis

Pharmacological agents are classified into two groups, antiresorptive and anabolic agents. The main mechanism of action of antiresorptive agents is reduction of bone resorption through inhibiting the activity of osteoclasts. Drugs of this class include calcitonin, bisphosphonates, estrogen, selective estrogen-receptor modulators, and denosumab. Administration of anabolic agents can result in new bone formation through stimulating the function of osteoblasts.

These medications may have some beneficial effects on extra-skeleton tissues and organs but by considering the most part of the burden of osteoporosis which is related to fractures especially hip fracture; the current approach to treatment of osteoporosis is focused on patient’s BMD and fracture risk. The beneficial effects of the pharmacological agents were presented in Table 2.

**Table 2:** Summary of characteristics of pharmacological agents for treating osteoporosis in clinical trials’ studies

Drugs class	Medication	Route of administration	Evidences of main effect		Ref.
			Increase in BMD	Decrease in fracture risk	
FDA/EU Approved					
Antiresorptive	*BPs*				
	Alendronate	PO	Spine 6.2%, hip 4.1%, LS 5.4%, FN 1.6%,	Vertebral 47%, hip 51%, non-vertebral 16%	[1, 33, 37]
	Risedronate	PO	Trochanter 3.3%	Vertebral 36%, non-vertebral 27%, hip 40%	[1, 37]
	Ibandronate	PO/IV	Nearly 6% for LS & hip	Vertebral 50%, Non-vertebral 30-40%	[1, 22, 34–37]
	Zoledronic acid	IV	LS 3.2%, FN 24%	Vertebral 70%, non-vertebral 25% (including hip 40%)	[37, 38]
	Clodronate	PO/IM/IV	LS 3.7%, hip 1.3%	Vertebral 43%, non-vertebral 33%	[22]
	*Denosumab*	SC	LS 9.2-18.4%, hip 4-8.3%	Vertebral 68%, hip 40%, non-vertebral 20%	[45, 46]
	*Estrogen Replacement* (ERT, HRT)	PO	LS 7.6%, hip 4.5%	ERT: Vertebral 38%, hip 39% HRT: Vertebral 35%, hip 33%, wrist 29%	[22, 52, 53]
	*SERMs*				
	Raloxifene	PO	LS 1.8%, hip 2.1%	Vertebral 35-43%, non-vertebral 10%	[22, 48, 57]
	Bazedoxifene	PO	LS 2.2-2.7%, hip (-1.15)-1.5%	(dose dependent) Vertebral 37-42%, non-vertebral no effect to 44-50% reduction	[56, 59, 60]
	Bazedoxifene+ conjugated estrogen	PO	LS 0.5-1.6%, hip 0.5-1.5%	NA	[62]
	Tamoxifene	PO	LS 1.2%	Overall 32% (hip 32%, spine 25%, radius 31%)	[63]
	Lasofoxifene	PO	LS 1.8-3.0%, hip 1.3-1.9%	(dose dependent) Vertebral 31-42%, non-vertebral 22-24%, no effect on hip fracture	[64, 66]
	Arzoxifene	PO	LS 2.75-2.9%, hip 1.53%	Vertebral 41%, no reduced non-vertebral	[67, 68]
	*Calcitonin*	Nasal spray/SC/IM	LS 1-1.5%	Vertebral 60%	[55]
Anabolic agents	*PTH peptides*				
	Teriparatide	SC	Spine 8.6-13%, FN 3.5-6%	Vertebral 65-69%, non-vertebral 53%	[6, 55, 72, 73]
	PTH 1-84	SC	LS 6.9%, FN 2.5%	Vertebral 60%	[11, 73]
Newer Agents (awaiting FDA/EU approval)					
Antiresorptive	*Cathepsin k inhibitors*				
	Odanacatib	PO	Spine 11.9%, total hip 8.5%, FN 9.8%	Hip 47%, non-vertebral 23%, clinical vertebral 72%	[71, 76]
	ONO-5334	PO	Spine 3.7-5.1%, total hip 3%, FN 2.6%	NA	[78]
	*Strontium*	PO	Dose dependent: LS 2.39-5.44%, FN 2.52-8.25%, total hip 1.02-8.25%	Vertebral 37-40%, non-vertebral 13%, hip 5%	[81, 82]
Anabolic agents	*Anti-sclerostin antibodies*				
	Romosozumab	SC	Dose dependent: LS 5.4-11.3%, total hip 4.1%, FN 3.7%	NA	[84]
	Blosozumab	SC/IV	Dose dependent: spine 8.4-17.0%, total hip 2.1-6.3%, FN 2.7-6.3%	NA	[85]
Combination therapy					
Antiresorptive and anabolic agents	*BPs + PTH*				
	Alendronate + Teriparatide	PO/SC	LS 14.8% vs. 18.1% by PTH/ 7.9% by BPs, total hip without differences	NA	[73]
	Risedronate + Teriparatide	PO/SC	Total hip 3.9% vs. 0.3% by PTH/ 0.8% by BPs, FN 8.4% vs. 3.9% by PTH/ 0.5% by BPs	NA	[73, 93]
	Zoledronic acid + Teriparatide	IV/SC	LS 7.5% vs. 7.0% by PTH/ 4.4% by BPs, total hip 2.3% vs. 1.1% by PTH / 2.2% by BPs	NA	[93]
	*Denosumab +* *Teriparatide*	SC/SC	LS 9.1% vs. 6.2% by PTH/ 5.5% by denosumab, total hip 4.9% vs. 0.7% by PTH/ 2.5% by denosumab, FN 4.2% vs. 0.8% by PTH/ 2.1% by denosumab	NA	[94]
	*SERMs+ Teriparatide* Raloxifene + Teriparatide	PO/SC	LS 6.2% vs. 5.2% by PTH, total hip 2.3% vs. 0.8% by PTH, FN 2.2% vs. 1.0% by PTH	NA	[57]
Antiresorptives	*BPs+ HRT*				
	Etidronate + Estrogen	PO/PO	LS 10.4% vs.7.0% by HRT, hip 7.0% vs. 4.8% by HRT	NA	[95]
	Alendronate + HRT	PO/PO	LS 10.1% vs. 4.0% by HRT, FN 4.0% vs. 2.0% by HRT	NA	[35, 96]
	Risedronate + HRT	PO/PO	LS 5.2% vs. 4.6% by HRT, FN 2.7% vs. 1.8% by HRT	NA	[35]
	*BPs+ SERMs* Alendronate + Raloxifene	PO/PO	LS 5.3% vs. 4.3% by BPs/ 2.1% by raloxifene, FN 3.7% vs. 2.7% by BPs/ 1.7% by raloxifene,	NA	[57, 95]

Legend: *BPs* Bisphosphonates, *FDA* Food and Drug Administration, *EU* Europe, *PO* oral route, *IV* intravenous, *SC* subcutaneous, *IM* intramuscular, *BMD* bone mineral density, *LS* lumbar spine, *FN* femoral neck, *GI* gastrointestinal, *HRT* hormone replacement therapy, *SERMs* selective estrogen receptor modulators, *AF* arterial fibrillation, *NA* no evidence available

#### Approved FDA/Europe antiresorptive drugs

##### Bisphosphonates (BPs)

BPs are recommended as the first-line medications for treatment of osteoporosis. Their effects on bone cells are most notable through inactivating osteoclastic bone resorption and accelerating apoptosis of osteoclasts. BPs can increase BMD, and decrease fracture risk. Drugs of this group include alendronate (Fosamax®), risendronate (Actonel®), ibandronate (Boniva®), zoledronic acid (Reclast®), clodronate (Bonefos®, Clasteon®), minodronate (Onobis®), pamidronate (Aredia®), etidronate (Didronel®), and tiludronate (Skelid®) which are different in terms of structure, potency, and affinity to bone. In addition, some of them such as etidronate and pamidronate are available in the US but not approved for prevention or treatment of osteoporosis [22].

Alendronate and risendronate are the most commonly used BPs worldwide. Alendronate not only has high affinity to bone, but also its effects have longer duration. Initiation of protective anti-fracture effect of alendronate is varied based on bone sites; 12, 18, or up to 24 months after treatment for vertebral bone, hip, and non-vertebral bone, respectively. Risendronate has a low affinity to bone, and its protective anti-fracture effect is started at least 6 months after treatment for vertebral and non-vertebral bones [32].

However, an increase in spine BMD rather than decrease in fracture risk was shown for zoledronic acid [33]. Clodronate is a weak BPs with beneficial effects on spine and hip BMD as well as vertebral and non-vertebral fracture risk in clinical trials. Its usage for osteoporosis has been approved in Europe [22]. A network meta-analysis compared the short term efficacy of different BPs including alendronate, clodronate, ibandronate, minodronate, pamidronate, risedronate, zoledronic acid, etidronate, and tiludronate in prevention of fractures in primary osteoporosis. The most effective BPs in the prevention of fracture at any sites was zoledronic acid, but alendronate or zoledronic acid revealed the highest effectiveness solely in preventing hip fracture [34]. Overall, the choice of BPs depends on the tolerance, cost, and medical history of patients. The influential effect of BPs in BMD and fracture risk varies in different studies. Some of the reasons are related to the age and sex of studied population, pre- or post-menopausal status, history of previous fracture, the type of the study (observational or clinical trial), and their comparison with placebo or other pharmacological treatments of osteoporosis [1, 22, 34–39]. A summary of the evidence-based data are shown in Table 2.

According to the American Association of Clinical Endocrinologists (AACE), response to treatment (not only with BPs but also with other pharmacological agents for treating osteoporosis) can be monitored by serial assessment of BMD (hip and lumbosacral) every one-two years after initiating BPs therapy until change in BMD become stable, and then repeats every two years or at less frequent intervals [23]. In some situations that absorption or efficacy of the drugs may be negatively affected, measuring bone turnover markers is recommended as an additional option; urinary N-telopeptide (NTX) and serum carboxy-terminal collagen crosslink (CTX) before and 3-6 months after initiating BPs and other antiresorptive agents. The extension of HORIZON-PFT trial and the Fracture Intervention Trial Long-Term Extension (FLEX) study revealed the highest beneficial effects of zelodronate after 3 years and alendronate after 5 years with regard to the fact that BMD and fracture outcomes were retained 6-9 years or 10 years after interrupting treatment with zelodronate or alendronate, respectively [40–42]. Thus, BPs therapy can be recommended beyond 5 years for patients at high risk of fracture. If the clinician decides to discontinue the treatment, it is necessary to assess BMD annually or biannually, concomitant to the measurement of bone markers and evaluating their changes. Hypocalcaemia, vitamin D deficiency, and renal dysfunction should be evaluated prior to initiating BPs through measuring serum level of Ca, 25-hydroxyvitamin D, and creatinine, and also the comorbidities that can influence on BPs usage and absorption should be assessed [35]. Some of these comorbidities are history of esophageal disorders such as achalasia that should be assessed before initiating oral BPs [35]. For achieving good efficacy of BPs, it is necessary to maintain optimal levels of calcium and vitamin D by adequate intake and/or supplementation. The most commonly reported adverse effects for oral BPs are gastrointestinal (GI) disturbances especially dyspepsia and esophagitis. So, oral BPs should be taken with an 8-ounce glass of water, upright for at least 30 minutes, and avoidance of sucking or chewing tablets. Flu-like symptoms was shown by taking large doses of oral or intravenous (IV) modes of BPs that can often be managed with acetaminophen. Other reported adverse effects are rare and include jaw osteonecrosis, atypical subtrochanteric femoral fractures, arterial fibrillation, and acute renal failure [35, 43–45]. Despite case reports of a serious adverse reaction by BPs [41], they have remained as the front line treatment for osteoporosis, at this time, based on risk-benefit ratio for using BPs especially reduction of the fracture risk [23, 35].

#### Denosumab

Denosumab (Prolia®) is a human monoclonal RANKL antibody that results in osteoclast inactivation, apoptosis, and reduction in osteoclasts’ differentiation by blocking the binding of RANKL to RANK. In addition a reduction of bone resorption as well as more than 80% decrease in serum level of CTX-1 has been reported [1]. Although it is not as the first-line pharmacological treatment of osteoporosis, but can be initiated as a first-line choice for treatment of osteoporosis in certain patients who are intolerant to oral BPs or have renal failure [46]. The “Fracture REduction Evaluation of Denosumab in Osteoporosis every 6 Months” (FREEDOM) trial showed the efficacy of denosumab on fracture-risk reduction at different skeletal sites among osteoporotic women [47]. Moreover, in patients at high fracture risk its beneficial effects were shown in older men under androgen-deprivation therapy for prostate cancer, and women who receive adjuvant aromatase inhibitor for breast cancer. Generally, it is not recommended to be used in premenopausal women or children and for prevention of osteoporosis. Its combination with other pharmacological agents for osteoporosis is not suggested. Similar to BPs, hypocalcaemia and vitamin D deficiency should be managed before starting treatment, and adequate Ca and vitamin D should be administered during treatment with denosumab. It was reported that the beneficial effects of denosumab on bone initiate after one month and maintain at least 2 years in different clinical trials [23, 48, 49]. Monitoring of response to treatment with BMD is similar to monitoring of other pharmacological agents for osteoporosis that BMD evaluation is recommended 2 years after treatment. Due to the expression of RANK and RANKL on T lymphocytes, B cells, and dendrites’ cells, an increase in the risk of infection by denosumab or its combination with other biologic agents is expected. Although there is no sufficient supportive data for infection or cancer in clinical trials, longitudinal studies are recommended to reveal the effect of long-term disruption in the receptor sites of RANK on immune system [48]. However, due to more frequent reports of serious infection and skin reaction with denosumab compared than placebo, patients should be informed to use appropriate drugs if develop signs of infection or skin reaction. Denosumab was not only well tolerated in osteoporosis clinical trials, but also no account of jaw osteonecrosis, arterial fibrillation, and symptomatic hypocalcaemia is reported, yet. Of the most commonly reported adverse effects were musculoskeletal pain, hypercholesterolemia, and cystitis [46].

#### Estrogen replacement

Due to the important role of estrogen deficiency on bone loss during menopause, it is suggested that use of estrogen replacement therapy (ERT) or estrogen-progestin (hormone) replacement therapy (HRT) alone is effective for prevention of osteoporosis in postmenopausal women [50]. Although the main effect of estrogen on bone health is reducing bone resorption, an anabolic effect was shown, too [51]. The Women’s Health Initiative (WHI) and the Postmenopausal Estrogen/Progestin Intervention (PEPI) trial showed beneficial effects of either ERT or HRT at all skeletal sites concomitant with some adverse effects in long term usage [52–54]. Tibolone is an estrogen-progestin combination that is available outside of the US. It is formulated as tablet and is commercialized under different brand names around the world, for example as Boltin® or Tibocina® in Spain and Xyvion® in Australia. Tibolone is used for prevention of osteoporosis and treatment of vasomotor symptoms of menopause. The Long-term Intervention on Fractures with Tibolone (LIFT) study conducted in post-menopausal women was shown its beneficial effects on reduction of fracture risk at vertebral (45%) and at non-vertebral (26%) sites [55]. It was shown that HRT can increase the risk of venous thromboembolic disorders, breast cancer, cardiac event and stroke, while ERT could enhance the risk of venous thromboembolic disorders, stroke, and endometrial cancer [23, 56]. Thus, estrogen replacement is recommended at the lowest effective dose and just for a short period. Moreover, HRT or ERT are not recommended as the first-line preventive treatment of osteoporosis. Beneficial effects of estrogen therapy on BMD will be decreased as nearly 5% during the first year after stopping treatment [22, 23].

#### Selective estrogen receptor modulators (SERMs)

Due to the adverse effects of estrogen in extra-skeletal organs, SERMs has been considered for treating osteoporosis in both sexes. SERMs contain nonsteroidal synthetic compounds with similar effects of estrogen on bone and cardiovascular system without any adverse effects of estrogen on breast and endometrium. This group includes raloxifene (Evsita®), tamoxifene (Soltamox®), lasofoxifene (Fablyn®), and bazedoxifene (Viviant®, Conbriza®).

Raloxifene is the first produced drug of this class. Its beneficial effects on bone were shown in the Multiple Outcomes of Raloxifene Evaluation (MORE) trial and the Raloxifene Use for the Heart (RUTH) study as reduction in vertebral fracture risk without any significant effect on non-vertebral fracture risk [57–59]. The safety and efficacy of raloxifene on BMD can be extended for eight years according to clinical trials. Although some clinicians are continuing its usage after 8 years, no residual benefit was shown on BMD after stopping usage [57]. Due to reduction of BMD and enhancement of bone turnover associated with raloxifene in premenopausal women, it is not recommended in this population. Raloxifene can decrease the risk of breast cancer, but can increase the rates of stroke, thromboembolism, leg cramp, and postmenopausal vasomotor symptoms [49]. So, new drug discovery efforts should be focused on new versions of SERMs with maximum beneficial effects on bone and the least adverse effects.

Bazedoxifene that is available in Europe and Japan have similar effects to raloxifene in osteoporosis [60, 61] but its long term safety or its impact on the risk of breast cancer is not yet determined. Although it is approved by Europe, is not approved yet by FDA at the time being. One formulation of bazedoxifene is its combination with conjugated estrogen (Duavee®) that can be used for treatment of osteoporosis and reduction of postmenopausal hot flashes [62]. Its common adverse effects are muscle cramps, GI disturbance, dizziness and neck pain. Other adverse effects are increased risk of stroke and thromboembolic diseases. Overall, the long term safety and preventive effect of Duavee on breast or ovarian cancers are unknown.

Tamoxifen as another drug from SERMs class has shown BMD reduction in premenopausal women by suppressing estrogen action on bone which is in contrast to its effect on postmenopausal women. But its impact on prevention or treatment of breast cancer in premenopausal is supportive. Thus, bone health can be monitored by evaluating BMD in this population [57, 63, 64]. Previous studies have reported that tamoxifen increases or stabilizes bone density, and decreases fracture rate in postmenopausal patients; however, no persisting benefit in terms of BMD is reported after 2 years of tamoxifen consumption following aromatase inhibitors treatment such as latrozole in postmenopausal women with estrogen receptor-positive breast cancer. Stopping tamoxifen would result in a rapid fall in estrogen levels induced by the aromatase inhibitors and may cause an accelerated BMD loss following the switch [65].

Due to the positive association between usage of tamoxifen and risk of endometrial hyperplasia or cancer, and vaginal bleeding, it should not be considered as the first-line therapeutic option of SERMs class for treating osteoporosis [57].

Lasofoxifene has shown protective effects on bone unlike its effect on breast and uterine. In different clinical studies conducted in postmenopausal osteoporotic women, some dose dependent beneficial and adverse effects of lasofoxifene at the dose of 0.25, 0.5, or 1.0 mg/ once daily were shown versus placebo [66, 67]. Lasofoxifene at the dose of 0.5 mg/daily was associated with reduction in vertebral and non-vertebral fracture risk, estrogen positive breast cancer, cardiovascular diseases (CVD), stroke, and increasing risk of thromboembolic events compared with the dose of 0.25 mg/daily [67]. It is approved by Europe but not approved by FDA.

Arzoxifene (LY353381) as a long acting raloxifene and ospemifene (Ophena®) that is structurally tamoxifene-like are new versions of SERMs with beneficial effects on bone such as increased BMD, decreased bone turnover, and fracture risk [49, 64, 68]. It should be noticed that clinical studies with ospemifene and arzoxifene are proceeding that are not approved by FDA, yet.

Generally, BPs or raloxifene are suggested as first-line choices for prevention of osteoporosis in postmenopausal women, pointing out that BPs are recommended over SERMs for treatment of osteoporosis. One should consider that the non-skeletal effects of SERMs have important role in selection of patients to use them. Raloxifene is contraindicated for prevention or treatment of osteoporosis in premenopausal women. BMD monitoring is required in the population taking tamoxifen [57].

#### Calcitonin

Calcitonin (Fortical®, Miacalcin®, Calcimar®) as a natural 32-amino-acid peptide is secreted by C-cells of the thyroid. It is considered as a second-line therapy for osteoporosis in settings where first-line drugs have failed to response or patients are found intolerable. Calcitonin is administered in two dosage forms; injectable and nasal spray that bioavailability of nasal calcitonin is about 1/4 that of its intramuscular route [69]. Usually, human calcitonin and salmon calcitonin have studied in clinical trials. Salmon calcitonin is used widely than human calcitonin due to its high affinity as 40 times more than human calcitonin for human calcitonin receptor. Calcitonin inhibits bone resorption through increasing osteoblast activity that results in reduction in vertebral fractures with a mild increase in spine BMD. Data on the effect of calcitonin on BMD of other skeletal sites are conflicting. However, its beneficial effects on bone were shown in both sexes by using both human and salmon calcitonin, both subcutaneous and intranasal routes [11, 69]. There are not sufficient data on its effectiveness in women who are in the early postmenopausal years. Thus, it is recommended as a second-line treatment for women at more than 5 years after menopause. Due to some reasons, including lesser effectiveness of calcitonin than BPs, increased risk of cancer by its long-term usage, and less availability compared to BPs, calcitonin is typically not used for treating osteoporosis unless to relief acute pain (onset <10 days) secondary to osteoporotic fracture. In contrast, calcitonin is not effective for chronic pain (more than 3 months). The adverse effects include nausea, vomiting, flushing, allergic reactions, hypocalcaemia, nasal adverse reactions, calcitonin’ antibodies formation, and prostate cancer. In required situations, one should cease its use in less than 6 months. In addition, after relieving from acute pain of fracture, calcitonin should be quickly switched to other pharmacological treatments of osteoporosis [69]. Calcitonin has withdrawn from the market in Europe and Canada. Although it is available in the US for treating osteoporosis, FDA Advisory Committee has not recommended it, yet [55]. In 2013, FDA advisory panels have recommended that marketing of calcitonin salmon for the treatment of osteoporosis in women greater than 5 years after menopause should be stopped. This is while in 2012, the European Medicines Agency had recommended that calcitonin salmon should not be used to treat osteoporosis after determining that the risk of developing cancer was 2.4% higher in patients using the nasal spray compared with in those who took placebo [70].

#### Approved FDA/Europe anabolic agents

##### PTH

Teriparatide (recombinant human parathyroid 1-34) (Forteo®) and intact molecule (amino acids 1-84) are peptides of PTH that have anabolic effects on bone mass and skeletal architecture by intermittent administration. Once daily subcutaneous administration of both agents have been shown to promote bone formation through activating osteoblasts’ function by binding to PTH/PTHrP type 1 receptor and stimulation of Wnt signaling pathway which resulted in increasing BMD and reducing fracture risk [71]. Both analogues can reduce vertebral fracture risk, but teriparatide can reduce non-vertebral fracture risk concomitant with reduced BMD in forearm [72]. A meta-analysis study showed a reduction in the risk of back pain due to reduced occurrence of new painful vertebral fractures [73]. Abaloparatide (BA058®) is a synthetic peptide analog of PTHrP that is in the process of applying to be approved in FDA and Europe. An increase by 6.7% was shown in a phase II clinical trial at lumbar spine BMD without significant difference from teriparatide and 2.6% increase at total hip BMD by abaloparatide with a significant difference from teriparatide. A phase III clinical trial concluded the reduction of major osteoporotic fracture risk by applying both formulations but significantly marked the administration of abaloparatide [74]. The beneficial effect of abaloparatide was shown on wrist fracture, too. So, it seems that the abaloparatide is superior to teriparatide in terms of fracture prevention [74]. PTH analogous are not recommended as first-line drug given to the cost of PTH analogous, subcutaneous (SC) route of administration, and the availability of other pharmacological agents for treating osteoporosis [73]. On the other word, these drugs are suitable for patients at high risk of fracture, severe osteoporosis (BMD < -3.0 T score), unsatisfactory response to anti-resorptive agents or unable to tolerate BPs, and patients with glucocorticoid-induced osteoporosis [6]. Its common adverse effects include dizziness, headache, nausea, and leg cramp [75]. Osteogenic sarcoma was shown followed by treatment with teriparatide in a dose and duration dependent pattern in experimental studies; however, it was not found in patients who used very trivial dosages of teriparatide [22]. Its use is contraindicated in patients with Paget, past bone irradiation therapy, hypercalcemia, hyperparathyroidism, bone metastasis, unexplained high level of alkaline phosphatase, and severe renal failure. It should be used with caution in patients with past or present history of kidney stones. Before the initiation of PTH therapy, BMD (if not assessed during two years ago), serum level of Ca, phosphorus, creatinine, alkaline phosphatase, albumin, 25-hydroxy vitamin D, and 24-hour urine excretion of Ca and creatinine should be evaluated. Vitamin D deficiency should be managed with vitamin D pretreatment. In addition, it is recommended to intake 1500 mg daily Ca and 800 IU daily vitamin D while receiving PTH. Transient orthostatic hypotension was reported in some cases. Overall, teriparatide treatment is limited to 2 years in a patient's lifetime. Usually, monitoring of BMD is not recommended during the first to the second year of PTH therapy, but renal function and serum Ca measurements should be performed at least once during treatment with PTH [73]. The beneficial effect of PTH on fracture risk has persisted for at least 18 months (up to 30 months for teriparatide) after stopping treatment which can be avoided by taking an antiresorptive agent such as BPs [55].

### New agents

The main goal of treating osteoporosis is based on marked increase in BMD and fracture free-period. Despite that current medications have effectively reduced fracture risk and increased BMD, understanding the targets of signaling pathways have helped to discovery of newer agents. Currently progression in development of newer agents such as cathepsin k inhibitor, strontium ranelate, AMG785, and AMG167 not only have increased the available options for treating osteoporosis, but also have opened doors of opportunity to improvements in the effective treatment.

#### Cathepsin k inhibitor

Cathepsin k is a cysteine protease that is expressed by osteoclasts. Cathepsin k can degrade matrix proteins and type I collagen that results in bone resorption. Cathepsin k inhibitors such as odanacatib (MK-0822, MK-822), balicatib (AAE581), and ONO-5334 can reduce bone resorption, and bone formation. Beneficial effect of odanacatib in the interim analysis of a phase III clinical trial (The Long-term Odanacatib Fracture Trial, LOFT) with postmenopausal women has shown moderate reduction in bone resorption (50%), lesser decrease in bone formation (30%) concomitant with beneficial effect on hip and spine BMD. The beneficial effects of odanacatib as a selective cathepsin k inhibitor on BMD were reported to be dose dependent and persist up to 5 years by treatment. In addition, its fracture risk reduction effect is comparable with the effects of BPs and denosumab on similar bone sites [76]. Results of a number of phase II clinical trials have shown its beneficial effect on bone resorption markers in a dose dependent manner in both sexes [77, 78]. A phase II clinical trial by AAE581 on site of ClinicalTrials.gov was found; however, the authors have not revealed any report of their study. Phase II OCEAN clinical trial demonstrated that ONO-5334 can decrease bone resorption similar to alendronate, with little or no change in bone formation and can increase BMD of lumbar spine, total hip and femoral neck comparable to alendronate [78]. However, its safety is not yet identified. All of these effects are reversible after stopping treatment. So, another medication should be added after taking off cathepsin k inhibitors to prevent loss of their beneficial effects on bone. Plaque-like skin thickening (morphea) that was one of the main adverse effects of early cathepsin k inhibitors (balicatib), was reported in 0.1% of users with odanacatib, ONO-5334 and other under development cathepsin k inhibitors (similar to placebo group). Some of the reported adverse effects of odanacatib versus placebo are trivial increase in the risk of stroke (1.4% vs. 1.1%), arterial fibrillation (1.1% vs. 1.0%), and atypical fractures (0.1% vs. 0%). However, these types of drugs are still under development and await approval [71, 79].

#### Strontium ranelate

Strontium ranelate (Protelos®) is an antiresorptive agent approved in Europe for treatment of severe osteoporosis in mobile postmenopausal women at high risk of vertebral and hip fractures who cannot use or tolerate other pharmacological agents. In addition, it is recommended for use in Europe to treat osteoporosis in men at high risk of fracture. Although its mechanism of action is unclear, modest antiresorptive effect and little beneficial effect on bone formation are noted. Inhibition of osteoclasts’ function as well as promotion of osteoblasts’ activity through calcium sensing receptor (CaSR) by strontium resulted in increasing BMD and decreasing fracture risk [80–82]. In addition, its effects on osteoblast differentiation and proliferation have been shown. Since the replacement of Ca ions in the hydroxyapatite crystals by strontium salt leads to a larger apparent increase in BMD, one should consider to include correction factors in BMD data of the strontium users to make up the effects of this artifact which may influence DXA results. The magnitude of changes observed in BMD followed by strontium therapy is not suggestive of a greater reduction in fracture risk. On the other word, a greater BMD doesn’t imply a larger reduction in fracture risk [27]. The more common reported adverse effects are cardiovascular events, venous thromboembolism, myocardial infarction, GI discomfort, and signs and symptoms of nervous system such as headache, seizure, memory loss, but rarely reported adverse effect is allergic reactions such as drug rash with eosinophilia and systemic symptoms (DRESS syndrome) [55, 77, 81]. Continuation of treatment with strontium should be stopped when hypertension, angina, or DRESS syndrome develop. In 2013, the European Medicines Agency’s (EMA's) Committee for Medicinal Products for Human Use (CHMP) recommended a restriction in the use of the osteoporosis medicine Protelos/ Osseor (strontium ranelate), following an assessment of data showing an increased risk of serious heart attack, with no observed increase in mortality risk. The CHMP recommended that Protelos should only be used to treat severe osteoporosis in postmenopausal women or in men at high risk of fracture. Additional measures, including restrictions in patients with heart or circulatory problems, were also recommended to minimize the heart risks of the medicine [83].

#### Anti-sclerostin antibodies

Anti-sclerostin monoclonal humanized antibodies such as romosozumb (AMG785), blosozumab (AMG167), and BPS804 are osteocytes-derived Wnt signaling antagonists. Stress can stimulate osteoblasts to secret osteocytes as a mechanical signaling that results in decreasing the expression of sclerostin. On the other word, sclerostin prevents bone formation through suppressing the binding of LRP 5/6 to Frz on osteoblasts which decrease differentiation, function and survival of osteoblasts. Increasing bone mass at spine and hips in clinical trials was shown concomitant with increasing in bone formation markers and decreasing in bone resorption markers by romosozumb and blosozumab [84, 85]. Variations in both of these significant bone markers’ will return to baseline levels (in a dose dependent manner) after 12 months which confirms the existence of other pathways independent of sclerostin for decreasing mechanical strain in the skeleton. Another reason of this event may be related to reduction of osteoblast progenitors or the compensatory activation of other signaling molecules such as DKK that reduce bone formation [86]. In addition, a positive association between bone mass and circulating level of sclerostin was shown in healthy men and women in contrast to the observed results in patients with bone disorders. The reason of this association may be related to high production of osteocytes as the main source of sclerostin secondary to higher bone mass [87]. So, more studies are needed to definite optimal doses, appropriate duration of treatment, and anti-fracture efficacy. The beneficial effects of anti-sclerostin antibodies were shown in animal models of other conditions resulted in decreasing BMD such as colitis, rheumatoid arthritis, osteoarthritis, osteogenesis imperfecta, and bone complications of type 2 diabetes mellitus [88]. The most frequently reported adverse effects are elevated liver enzymes after first dose that is normalized nearly after one month, and injection site reactions. There are rarely reported anti-blosozumab antibodies that decline with stopping treatment. In addition, just one case of nonspecific hepatitis after taking 10 mg/kg of romosozumab was reported which improved after 26 days [71]. By considering the hypothesis that activation of Wnt signaling can increase cardiovascular events or intracranial pressure due to unrestricted bone formation, and also the relation between sclerostin and some tumors such as colon cancer, long-term safety of these drugs are uncertain.

#### Other therapies

There are additional therapies for postmenopausal osteoporotic women such as vitamin K, folic acid, vitamin B12 supplementations, androgens, and fluoride that are used in some countries, but are not recommended for routine use [27, 89]. However, there are other agents that have been shown to influence bone health through antiresorptive or anabolic mechanism, but are under investigations. The RGD sequence and human ß3 integrin have been reported as important targets for antiresorptive gene therapy. As a result, some studies have suggested the use of mesenchymal stem cells, which are osteoblast precursor cells, to carry the therapeutic gene. OPG, BMP and PTH have been reported as the most promising molecules for osteoporosis treatment, but not many new molecules have been studied as possible targets in this regard.

Various cytokines and cytokine antagonists (cDNA encoding the human interleukin-1 receptor antagonist (IL-1Ra) have also shown promising results as new therapeutic agents for osteoporosis in animal models, but their application is hindered by delivery problems. However, there are some barriers on use of these drugs for osteoporosis treatment that are as following [90, 91]:

- It was shown the calcified matrix of the bone tissue is considered as the main barrier in gene therapy for osteometabolic diseases, because the diffusion of the vector through such a matrix is practically impossible.
- Another obstacle in this regard is the use of single therapeutic genes in most studies, as a single gene cannot affect both the osteoblasts and the osteoclasts at the same time.

Overall, some of these new drugs are including frizzled-related protein inhibitor, glycogen synthase kinase 3 (GSK-3) inhibitors; lithium, 603281-31-8, 6-bromoindirubin-3^´^-oxime, AR28, matrix metalloproteinases (MMPs), selective androgen receptor modulators (SARMs), cell adhesion molecules (CAMs), L-carnitine and its derivatives, amylin, adrenomedullin, reveromycin A, insulin like growth factor-1 (IGF-1), and mesenchymal stem cells [18, 49].

#### Combination therapy

It is hoped that the combination therapy show synergistic and additive anti-osteoporotic effects. So, combined agents with different mechanism of action such as antiresorptive plus anabolic agents or combination of two or more agents with the same mode of action such as inhibitors of bone resorption can be considered as an appropriate strategy for treatment of osteoporosis. However, it should be noticed that the metabolism of a drug is varied individually, and the safety and efficacy of drugs would change according to individual differences [92]. The combination of BPs and PTH 1-34 or PTH 1-84 in several clinical trials resulted in reduction in anabolic effect of PTH. In the most of studies alendronate in combination with PTH was used. Although combination therapy improved BMD at spine or hip, there was not additional benefit on BMD compared with PTH therapy alone [73]. The reduction of bone resorption markers was found in the studies conducted on both sexes showing no increase in bone formation markers in combination group. Recently, it was shown in one small clinical trial in osteoporotic men that the combination of teriparatide and risendronate increased BMD at hip compared with either therapy alone. Although BPs plus PTH is not recommended for management of osteoporosis, immediate use of BPs after withdrawing teriparatide can increase BMD at lumbosacral. Combinations of teriparatide and denosumab, estrogen or SERMs were shown small additive effect on BMD [73, 93, 94]. However, it should be noted that these studies were not designed to assess the effect of the combination on fracture risk.

Although some studies reported the combination of estrogen and BPs more effective than each of them alone, this combination is not recommended, because of the over suppression of bone turnover and enhancement of fracture risk [95, 96]. Alendronate plus estrogen or raloxifene increased BMD to a greater extent when compared to either drug alone, in addition to unknown benefit for fracture reduction [35, 57]. Overall, combination of the above drugs are not suggested because of their small beneficial effect on BMD, no proven additional benefit on fracture risk, increasing in cost and potential adverse effects of the drugs [93].

Combination of anti-inflammatory agents such as anti-tumor necrosis factor (anti-TNF) with anti-sclerostin antibodies in animal models have been shown to be more effective than either agent alone. By this combination was observed prevention of cortical and trabecular bone loss, and restoration of vertebral bone to levels in wild-type mice who had not arthritis. In addition, significant reduction of osteoclast number, suppression of bone erosion to lower level than baseline, and significant increase in thickness, area, and proteoglycan content of cartilage were observed [97]. Thus, it is suggested that medication selection would be on an individual basis. Personalized medicine that includes personalized genetic risk score and individualized environmental exposure may have a key role in pathophysiology of osteoporosis [98].

### Osteoporosis in men

Due to the higher prevalence of osteoporosis among women than men, greater focus is paid on treatment of osteoporosis in women and several large randomized clinical trials have been conducted on this population. However, morality rate secondary to major fractures, especially of the hip, has increased in osteoporotic men than in women. Thus special attention should be paid to the treatment of osteoporosis in men. Similar approach is recommended in treating osteoporosis in both sexes. Osteoporotic men should intake adequate Ca (from 1000 to 1200 mg daily according to age) and vitamin D (from 600 to 800 IU per day) supplementation in addition to pharmacological or hormonal therapy. Because the most common cause of osteoporosis in men is hypogonadism, testosterone replacement therapy can increase BMD by 5% in spine. Data of clinical trials confirms the beneficial effect of testosterone on BMD for at least 2 years [99]. Androgens have beneficial effects on cardiovascular system and adverse effects on prostate [100]. Androgen replacement therapy beside other pharmacological agents for treatment of osteoporosis is recommended for hypogonadal men at fracture risk [101]. Although it is expected that androgen therapy should have beneficial effects in elderly men with osteoporosis, sarcopenia, or falls, a meta-analysis study did not show its beneficial effects on bone health [102].

Overall, all pharmacological agents can be safely considered as a therapeutic option in the treatment of osteoporosis in men [103]. Alendronate or risendronate are favorable BPs that are recommended as the first line pharmacological therapy for osteoporosis in men. Zoledronic acid is a suitable alternative in patients who do not tolerate oral BPs. Denosumab is a good choice in osteoporotic men who are intolerant to other drugs or have renal failure. BPs therapy should be stopped in men with severe osteoporosis who are intolerant or unresponsive to BPs therapy after one year, and PTH therapy should be selected as an alternative. As recommended for postmenopausal women, monitoring of treatment with BMD evaluation is of utmost importance [101].

### Recommendations for future research

It is not only required to conduct more studies on understanding the signaling pathway, but there are also needs to conduct more studies on prevention of osteoporosis, safety and efficacy of anti-osteoporotic pharmacological agents, and drug discovery. It was reported in most of the studies that antioxidative mechanism has the main role in prevention and treatment of osteoporosis [104–106]. Some of these products include green tea, quercetin, curcumin, phytoestrogens, omega-3 fatty acids and soy isoflavones. In addition, it was reported anti-resorptive effects for some active compounds such as flavonoids, terpenoids, glycosides, lignans, coumarins, alkaloids, and polyphenols, etc that have derived from medical plants [107–111].

Polyphenols that are found in the most plants has shown to be effective in increasing bone formation, decreasing bone resorption, and increasing bone strength. Epigallocatechin-3-gallate (EGCG) is the main polyphenol in green tea. Some of its beneficial effects on bone health are modulated through suppression of the transcription factors such as Runx2, or act through some molecular signaling pathways such as OPG/RANKL, mitogen-activated protein kinase (MAPK), and BMP that resulted in suppression of osteoclast differentiation, and affect osteoblast function [18]. Phytoestrogens can inhibit aromatase and cytochrome P450 by binding to estrogen receptors. High level of aromatase has positive association with risk of breast, adrenal and prostate cancers. Other mechanisms include the influence on calcium absorption and its urinary excretion, prostaglandin synthesis, osteoblast formation and lipid oxidation. The beneficial effect of phytoestrogens on bone resorption markers, and BMD in postmenopausal women in some meta-analysis of randomized controlled trials was reported, in contrast to others [112–115]. The effect of isoflavones on fracture risk was not seen. However, it should be noticed that phytoestrogens can be considered as the best alternative of estrogen therapy for relief of menopausal symptoms. Similar to synthetic drugs, it is hypothesized that more beneficial effects for polyherbal combination be revealed in the near future. The greens + ^TM^ a blend of several polyphenols including quercetin, apigenin and luteolin, combination of several types of omega-3 fatty acids, and Resveratrol in combination with vitamin D have shown beneficial effects on bone more than when used alone [18, 110, 116]. However, most of these studies have been performed in vitro and needs preclinical animal studies to optimize the dose with maximum beneficial effects on bone health, and then well-designed clinical studies with large sample sizes and long follow-up duration in at-risk populations.

## Conclusions

Currently, the development of pharmacological agents in treatment of osteoporosis has discovered new agents that effectively reduce fracture risk. Due to the lack of appropriate clinical trials for newer anabolic or antiresorptive agents, until these agents are approved, it is recommended to prescribe the current available agents that is more appropriate to each patient. Thus, the physicians should be aware about the current drugs and also newer agents’ mechanism of action, their beneficial effects on bone health, the best time to prevent fractures, optimal beneficial duration of treatment, and dosing requirement.

Some limitations exist despite the major advances in drug discovery for treatment of osteoporosis. First, their efficacy on hip fracture reduction is lower than what observed on lumbosacral fracture. Secondly, potential adverse events may emerge by using anti-osteoporotic pharmacological agents in postmenopausal osteoporotic women who need long term treatment. Thirdly; the benefits associated with so called treatment strategies are limited to selected patients, generally those at higher risk of fracture. However, monitoring of complications in long-term may raise some concerns. Forth, due to the high cost of new agents, their usage should be restricted in selective patients who are at high risk of fracture or when failed response to first line treatment options. In addition, individuality in the metabolism of a drug affects drug safety and efficacy. On the other hand, the progressive advances in the personalized therapy for osteoporosis raises the necessity for identifying the main genes and signaling pathways involved in bone loss of individual patients. Henceforth, the effects of these genes and pathways modulate the application of therapies in every specific individual. Thus, personalized medicine should be considered for genetic risk score and also for environmental exposure assessment.

In addition to continuous attention to the early diagnosis of osteoporosis, more well-designed clinical trials is required on the safety and efficacy of current available anti-osteoporosis agents. As well, continuous preclinical assessment of newer agents, conduction of clinical trials, searching for novel approach in drug discovery based on understanding of the pathophysiology of osteoporosis are strictly recommend. The authors also suggest conducting future research on plant-derived components as the source of discovery of new agents, and also more clinical trials with combination of two or more synthetic drugs, plants, or drug-plant for treatment of osteoporosis as the solutions.

## Abbreviations

AACE: American Association of Clinical Endocrinologists
anti-TNF: anti-tumor necrosis factor
BMD: bone mineral density
BMP: bone morphogenetic protein
BPs: Bisphosphonates
Ca: calcium
CAMs: cell adhesion molecules
CaSR: calcium sensing receptor
CHMP: Committee for Medicinal Products for Human use
CTX: carboxy-terminal collagen crosslink
DEXA: dual-energy x-ray absorptiometry
DKK: Dickkopf
DRESS syndrome: drug rash with eosinophilia and systemic symptoms
EGCG: Epigallocatechin-3-gallate
EMA’s: European Medicines Agency’s
ERT: estrogen replacement therapy
ERα: estrogen receptor α
ERβ: estrogen receptor β
FDA: Food and Drug Administration
FLEX: Fracture Intervention Trial Long-Term Extension
FREEDOM: Fracture REduction Evaluation of Denosumab in Osteoporosis every 6 Months
Frz: frizzled receptors
GI: gastrointestinal
GSK-3: glycogen synthase kinase-3
HRT: estrogen-progestin replacement therapy
IGF-1: insulin like growth factor-1
IOF: International Osteoporosis Foundation
IU: international units
IV: intravenous
LIFT: Long-term Intervention on Fractures with Tibolone
LOFT: Long-term Odanacatib Fracture Trial
LRP5: LDL receptor-related protein5
MAPK: mitogen-activated protein kinase
MMPs: matrix metalloproteinases
MORE: Multiple Outcomes of Raloxifene Evaluation
NTX: N-telopeptide
PEPI: Postmenopausal Estrogen/Progestin Intervention
PTHrP: PTH-related protein
RANK: receptor activator of NF-κB
RANKL: RANK ligand
ROS: reactive oxygen species
Runx2: runt-related transcription factor2
RUTH: Raloxifene Use for the Heart
SARMs: selective androgen receptor modulators
SERMs: Selective estrogen receptor modulators
SFRP: secreted frizzled-related protein
WHI: Women’s Health Initiative
Wnt: wingless-type and integrase1
